# Efficacy of Synchronous vs. Asynchronous Telerehabilitation for Musculoskeletal Symptoms in Post-Covid-19 Syndrome: A Randomized Clinical Trial

**DOI:** 10.63144/ijt.2025.6716

**Published:** 2025-12-12

**Authors:** Nadine Carneiro Tura, Franciele da Silva Pereira, Bruna Fogaça, Anne Sofia Pang, Lívia Arcêncio do Amaral, Rafael Inácio Barbosa

**Affiliations:** Postgraduate Program in Rehabilitation Sciences, Federal University of Santa Catarina, Araranguá, SC, Brazil

**Keywords:** Asynchronous, Long COVID, Physical functional performance, Post-acute COVID-19 syndrome, Synchronous, Telehealth, Telerehabilitation

## Abstract

**Objective:**

Compare the effects of physiotherapist-supervised synchronous telerehabilitation (TR) with unsupervised asynchronous TR in adults diagnosed with post-COVID syndrome (PCS).

**Methods:**

In this single-blind randomized controlled trial conducted with 31 participants with PCS were randomized into a synchronous telerehabilitation (STR) group, which underwent two-hour sessions per week for eight weeks, and an asynchronous telerehabilitation (ATR) group, which performed unsupervised exercises. Lower limb functional strength (Five Times Sit-to-Stand Functional Test) as the primary outcome, and the dyspnea (Modified Medical Research Council), fatigue (Fatigue Assessment Scale), stress, anxiety, depression (Depression, Anxiety, and Stress Scale-21), and quality of life (World Health Organization Quality of Life-BREF Questionnaire) were assessed remotely at the baseline, after 8 weeks of intervention, and at a 20-week follow-up. Data were analyzed using a mixed-model analysis of variance.

**Intervention:**

Participants were randomized into a synchronous telerehabilitation (TRS) group, which performed two-hour sessions per week for eight weeks, and an asynchronous telerehabilitation (TRA) group, which performed the same exercise protocol but without the supervision of a physiotherapist. Instructional videos were made available via social media (WhatsApp and YouTube). Participants were also instructed to perform the protocol twice a week for eight weeks.

**Results:**

A statistically significant difference was only observed in lower limb functionality between both groups (p = 0.02). The STR group demonstrated significant improvements in lower limb functional strength (p = 0.03), dyspnea (p = 0.02), fatigue (p = 0.00), stress (p = 0.03), and quality of life (p = 0.00), without any adverse events. Conversely, the ATR group experienced significant improvements in fatigue (p = 0.00) and anxiety (p = 0.02).

**Conclusion:**

The present findings show that both modalities demonstrated positive effects over an 8-week TR program in adults with PCS. However, the synchronous approach achieved greater improvements in lower limb functionality, dyspnea, fatigue, stress, and quality of life. Our findings revealed that asynchronous model was associated with higher dropout rates and suggest synchronous TR may offer advantages regarding treatment adherence.

The global impact of the COVID-19 pandemic was substantial, resulting in persistent symptoms such as fatigue, dyspnea, loss of smell, as well as physical symptoms like joint pain and myalgia in individuals infected with the virus. These symptoms can last for more than 12 weeks and extend up to two years without proper treatment (Lopez-Leon et al, 2021; Wahlgren et al., 2023). Termed post-COVID syndrome (PCS) or long COVID, (CDC, 2023) this condition has been linked to reduced functional capacity and diminished quality of life (Tanriverdi et al., 2022). Joffe and Elliott (2023) defined PCS as a functional disorder that also affects mental well-being, highlighting the profound influence of pandemic-induced fear and uncertainty on individual symptoms. Considering these challenges, there is an urgent need to investigate effective treatment strategies for improving functional capacity and mitigating fatigue in individuals with PCS.

Telerehabilitation (TR), an offshoot of telemedicine, employs information technology for rehabilitation services (Smith et al., 2020; Valverde-Martínez et al., 2023). Its growth is linked to tech advancements, increased internet access, and COVID-19 measures. While established in high-income countries, it lags in low- and middle-income nations like Brazil due to limited readiness and awareness (Fernandes et al., 2022; Zheng et al., 2022). A recent meta-analysis conducted by Yang et al. (2024) demonstrated consistent effects of resistance training on different indicators of physical function in patients with patellofemoral pain syndrome (PFPS). The authors identified a significant improvement in muscle performance as assessed by the 30-second sit-to-stand test, with an average increase of 1.58 repetitions (6 RCTs, n=310; 95% CI 0.50–2.66; p=0.004). Furthermore, resistance training promoted a significant gain in aerobic functional capacity, evidenced by an increase of 76.90 meters in the distance covered in the 6-minute walk test (6 RCTs, n=324; 95% CI 49.47–104.33; p<0.00001). Self-reported physical function also showed improvement, with an average increase of 6.12 points in the physical component of the SF-36 questionnaire (5 RCTs, n=380; 95% CI 2.85–9.38; p=0.0002). These results reinforce that resistance training is an effective strategy for improving different domains of physical function in individuals with chronic pain syndrome.

However, the study also identified methodological shortcomings in current clinical trials, along with disparities in TR approaches, insufficient program implementation guidelines, and a deficiency in professional training. The positive outcomes of TR align with the findings of a systematic review on TR’s effectiveness in treating chronic respiratory diseases, supporting its viability as an alternative to in-person rehabilitation (Cox et al., 2021).

Rehabilitation therapy (RT) can be performed synchronously or asynchronously (Gustavson et al., 2021). In synchronous RT, patients and healthcare professionals interact in real time, resembling traditional consultations. Currently, it is possible to use educational and health methods, as well as technological tools that make the session more engaging (Zanaboni et al., 2017). This format fosters the development of a therapeutic alliance through active collaboration, a crucial element for effective therapy (Ferreira et al., 2013; Kinney et al., 2020). In contrast, asynchronous TR typically relies on pre-recorded exercise videos, written treatment plans, and digital platform or app-delivered educational materials, posing challenges in establishing a therapeutic alliance (Bini et al., 2017).

For instance, Tahan et al (2023) randomized clinical trial found no significant disparities between asynchronous and synchronous TR in PCS management over an 8-week span. However, the study did find more positive effects on physical function, emotional well-being, and anxiety in the synchronous format, highlighting the importance of the therapeutic alliance in PCS rehabilitation. To our knowledge, this is the only study that has compared both TR modalities in PCS, indicating a need for further research given the complexities of implementing TR in low- and middle-income countries. Indeed, this raises the question of whether the presence of a physiotherapist is more beneficial or whether patients can effectively manage their own rehabilitation.

Considering these factors, this randomized controlled trial (RCT) aimed to assess the short-term effectiveness of a physiotherapist-supervised synchronous TR functional exercise program compared to an asynchronous TR program without supervision. The objective was to enhance lower limb function, alleviate dyspnea, mitigate fatigue, stress, anxiety, depression, and enhance the quality of life in adults diagnosed with PCS in a low- and middle-income country. Our hypothesis was that participants in the group receiving synchronous TR would demonstrate greater improvement in lower limb function than those in the group that received asynchronous TR.

## Methods

### Study Design

This is a single-blind, parallel-group randomized clinical trial consisting of an exercise program offered by supervised synchronous and unsupervised asynchronous telerehabilitation associated with telehealth guidance, which lasted eight weeks, with two sessions per week, totaling 16 sessions included three evaluation sessions (baseline, week 8, and week 20 at follow up). This study was reported following the CONSORT-Outcomes 2022 extension of the 2010 Statement, the primary outcome was the Five Times Sit-to-Stand Functional Test (FTSST), while the secondary outcomes were the Modified Medical Research Council (mMRC) scale, fatigue assessment scale (FAS), Depression, Anxiety, and Stress Scale-21 (DASS-21) and the World Health Organization Quality of Life-BREF (WHOQOL-Bref).

### Recruitment and Eligibility Criteria

Individuals with persistent COVID-19 symptoms were consecutively screened and recruited through various sources, such as social networks, the local community, and different regions of Brazil. Recruitment methods included distributing city flyers, digital flyers on social media, and radio announcements. Interested individuals completed an online form providing identification, demographics, and selection information, followed by signing the informed consent form. This study was reported following the CONSORT-Outcomes 2022 extension of the 2010 Statement, approved by the Human Research Ethics Committee of the Federal University of Santa Catarina (no. 50003221.1.0000.0121) and registered at ensaiosclinicos.gov.br (no. RBR-8v2qmyz). Data collection was conducted entirely through an online form, without direct interaction between the researcher and participants, due to the prevailing health context. During this period, social distancing measures and restrictions imposed by the COVID-19 pandemic made in-person procedures unfeasible, making a remote approach the safest and most ethically appropriate methodological alternative. In this way, the online strategy ensured the continuity of the research, preserved the safety of those involved, and complied with the recommendations of the health authorities at the time. Inclusion criteria included being 18 or older, self-reported presence of at least one persistent COVID-19 symptom (e.g., fatigue, dyspnea, joint pain, or myalgia) and scoring 1-2-3 on the mMRC scale (Bestall et al., 1999; Casanova et al., 2015; Kovelis et al., 2008). Individuals were excluded if they recovered from COVID-19 without lingering symptoms, scores of 4–5 on the mMRC scale, absence from four or more telerehabilitation sessions and/or two consecutive sessions, and the inability to perform the proposed activities.

### Randomization, Blinding and Interventions

Participants were randomly assigned to either synchronous telerehabilitation (STR) or asynchronous telerehabilitation (ATR). An independent researcher created a randomized allocation list (www.randomizer.org) using 4 blocks of 12, 16, 11, and 5 participants each. A code assigned to each participant concealed their group assignment. The evaluator, consistent throughout the study, remained blinded to group allocation. The physiotherapist, however, was not blinded to group assignments.

Both groups received COVID-19 health education, covering prevention, disease management, and PCS implications. Participants refrained from other physical activities during the study. The exercise regimen included warm-up, aerobic, limb strengthening, joint mobility, chest stretching, and breathing exercises. The STR group exercised via video call with a physiotherapist, while the ATR group used only video instructions. Participant blinding was maintained, and they received no financial incentives. Group assignment was revealed after the study, with ATR participants having the option to perform the STR exercise program.

### Synchronous Telerehabilitation

The STR group engaged in an eight-week progressive exercise program comprising two sessions per week. The duration varied according to each patient, averaging 50 minutes. These sessions were remotely supervised by a physiotherapist via video conferencing, providing performance oversight, addressing questions, and helping participants with difficulties. The exercise program was divided into multiple elements (see Supplementary Material): a one-minute warm-up involving aerobic exercises, progressive lower and upper body strengthening exercises, trunk exercises, and chest stretches combined with breathing exercises. The warm-up phase lasted one minute, during which the Borg Rating of Perceived Exertion Scale was administered. A threshold value of 6 was set to maintain moderate activity levels (Helms et al., 2020). In the initial week, participants completed three sets of eight repetitions for each strengthening exercise, using their body weight, a 0.5-kg water bottle, or a 1-kg water bottle as resistance, with one-minute rest intervals. Repetitions increased every two weeks, reaching 16 by the protocol’s end (Aily et al., 2020; Gentil et al., 2021). Participants were advised to avoid any other physical activities during the study.

### Asynchronous Telerehabilitation

The ATR group performed the same exercise protocol as the synchronous group, but without any direct supervision from the physiotherapist. For this group, pre-recorded instructional videos were provided, containing planned predictions for each exercise, lasting approximately 30 minutes per session, sent via WhatsApp and an unlisted YouTube channel. These videos included general guidelines on execution, pace, and progression, but did not allow for real-time interaction. Participants were instructed to follow the protocol twice a week for eight weeks, without the possibility of asking questions, requesting adjustments, or obtaining individualized feedback after the sessions, as there was no active communication channel with the researchers.

Furthermore, it was not possible to directly document or monitor adherence to the sessions in the ATR group, which represents a significant methodological limitation, especially when compared to the synchronous group, which had access to real-time supervision and the opportunity to request help or clarification during practice. This difference in the possibility of interaction and support may have introduced bias in access to the intervention, potentially influencing the execution of the exercises and, consequently, the results observed between the groups.

### Evaluations and Outcomes

Participants from both groups underwent identical evaluations at three distinct time points: baseline, after the 8-week exercise program, and at a 20-week follow-up. The principal outcome measure was the time, in seconds, required to complete the FTSTS. This test was administered remotely via digital platforms last, after the other assessments had been completed (López-López et al., 2022). The required setting included a 3 or 4 m^2^ area of flat, non-slippery ground and a stable chair that allowed the participant’s feet to touch the floor while seated (Araújo et al., 1999). Fatigue perception was assessed using the FAS, which is commonly employed in research involving cardiorespiratory-related diseases (Michielsen et al., 2014; Moore et al., 2013). Neuropsychiatric symptoms were evaluated through the DASS-21, divided into stress, anxiety, and depression subscales (Imamura et al., 2021; Lovibond & Loovibond, 1995; Vignola & Tucci, 2014) Quality of life was assessed by WHOQOL-Bref instrument and evaluated in a 0–100 score (Fleck et al., 2000; Pedroso et al., 2010). Initially, on the RCT register, there was another scale under assessment, the Patient-Specific Functional Scale, which had to be excluded from data collection due to incorrect utilization and missing data.

### Sample Size

The sample size was estimated for the primary outcome, functional strength of the lower limbs as assessed by the FTSST. The calculation was made using the G*Power version 3.1 software, the calculation was done for a repeated measures analysis of variance, accounting for both intra- and inter-subject interactions, with an F effect size of 0.25, α of 0.05, and power (1-β) of 0.80, for two groups with three measurements each. This yielded a sample size (n) of 28 subjects, allocating 14 volunteers to each group. To account for a potential 20% attrition rate, two additional volunteers were included in each group, totaling 32 participants.

### Statistical Analysis

Intention-to-treat (missing data handled using the average imputation method) was performed for all outcomes. Data normality was tested using the Shapiro-Wilk (Shapiro; Wilk, 1965) and the efficacy of STR versus ATR over multiple assessment time points and any changes in scores were evaluated using a mixed model effects, followed Tukey pos-hoc test. The effect size was calculated using the eta-squared test for fixed variables, which yielded a Cohen ’s d value used to compare the proportion of participants in each group with a clinically important improvement. The level of significance was P<.05. All data analyses were conducted using GraphPad Prism 8.0.1 software.

## Results

**Figure 1:**
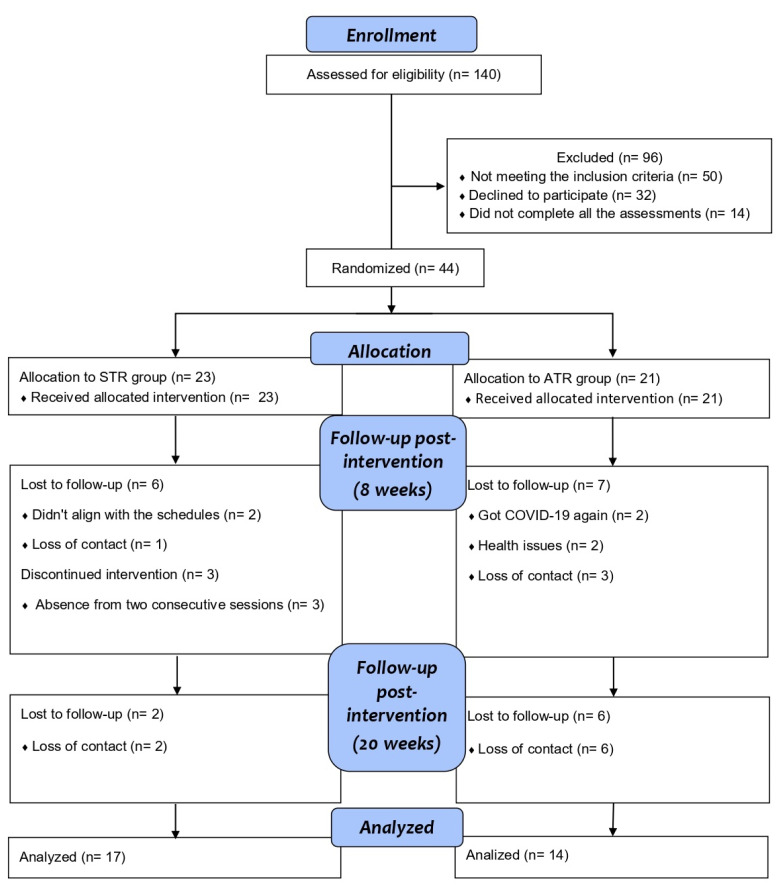
Flowchart of the Clinical Trial

Between March 2021 and March 2023, we successfully randomized 44 consecutive participants, with 23 assigned to the STR group and 21 to the ATR group. However, after the final twenty-week period, attrition occurred, resulting in the loss of 8 participants from the STR group and 13 from the ATR group during follow-up. Given the missing data at follow-up, we conducted an intention-to-treat analysis, which yielded a final participant count of 17 in the STR group and 14 in the ATR group. Two participants needed triceps exercise adjustments in the first 4 weeks in the STR group, highlighting a need for protocol refinements in future studies. A flowchart detailing the study ’s participant progression is illustrated in Figure 1 and, at baseline, demographic and clinical characteristics were similar for all the groups (Table 1).

### Primary Outcome

#### Lower Limb Functionality

**Figure 2:**
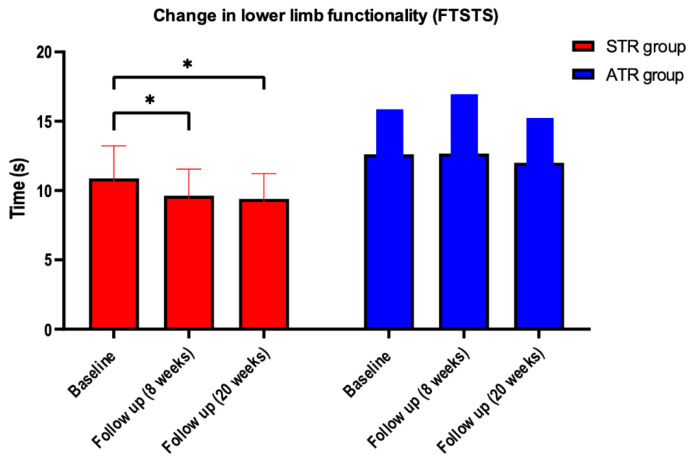

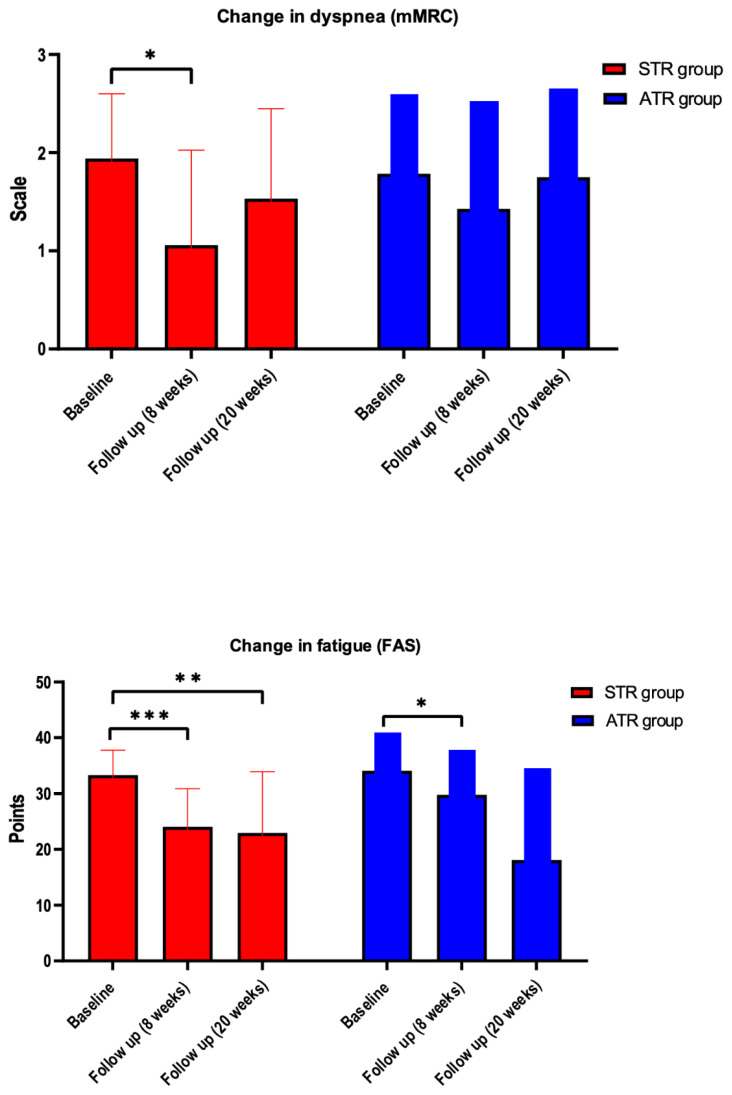

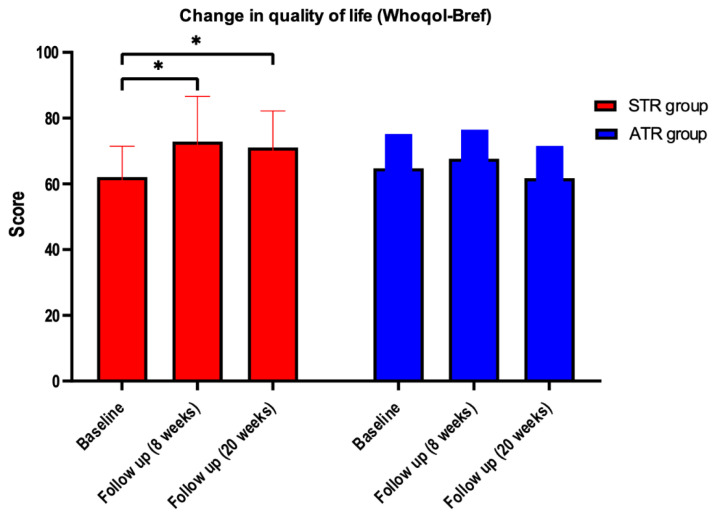
Change in Lower Limb Functionality Measured by FTSTS, Dyspnea Measured by mMRC, Fatigue Measured by FAS and Overall Quality of Life Measured by Whoqol-BREF Variables at Baseline, at 8 Weeks of Follow Up and During a Follow Up of 20 weeks *Note*. Changes whitin groups in patients allocated to STR group compared to patients allocated to the ATR group; *significant difference (p<0.05) within groups Tukey’s post hoc test; **significant difference (p<0.01) within groups Tukey’s post hoc test; *** significant difference (p<0.001) within groups Tukey’s post hoc test

A significant between-group difference (F = 5.936; p = 0.0269) was found in the FTSST by mixed-model effects, but in the post hoc, this difference was not found. The effect size of STR on FTSST was of medium magnitude (d = 0.51) between groups. In the intra-group analysis, a significant difference was also found (F = 5.606; p = 0.01) by the mixed models effects. The post hoc showed a significant difference between the initial assessment vs. eight weeks of intervention for the STR group (95% CI = 0.1004–2.396; p = 0.0291) and the initial assessment vs. 20 weeks of intervention for the STR group (95% CI = 0.06205–2.899; p = 0.0386). The effect size of STR on FTSST was of medium magnitude (d = 0.51) (Table 2 and Figure 2) within groups.[Table t3-ijt-17-2-6716]

### Secondary Outcomes

#### Perception of Dyspnea

There was no significant difference between groups, only intra-group (F = 6.192; *p =* 0.0123) on the mMRC scale. In the post hoc test, the difference was localized between the initial assessment vs. eight weeks of intervention in the STR group (95% CI = 0.1066–1.658; *p =* 0.0212) (Figure 2). The effect size of the intervention was of medium magnitude (d = 0.54) within groups.

#### Perception of Fatigue

There was no significant difference between groups but a significant intra-group difference (F = 21.01; *p* < 0.0001) in the FAS. In the post hoc analysis, the difference was located between the initial assessment vs. eight weeks of intervention for the STR group (95% CI = 4.197 to 14.27; *p =* 0.0003); initial assessment vs. 20 weeks of intervention for the STR group (95% CI = 2.504 to 18.20; *p =* 0.0067); and initial assessment vs. 20 weeks of intervention for the ATR group (95% CI = 2.519 to 29.47; *p =* 0.0173) (Figure 2). The effect size of the intervention on FAS was of large magnitude (d = 1.06) within groups.

#### Self-reported Stress, Anxiety, and Depression

**Figure 3:**
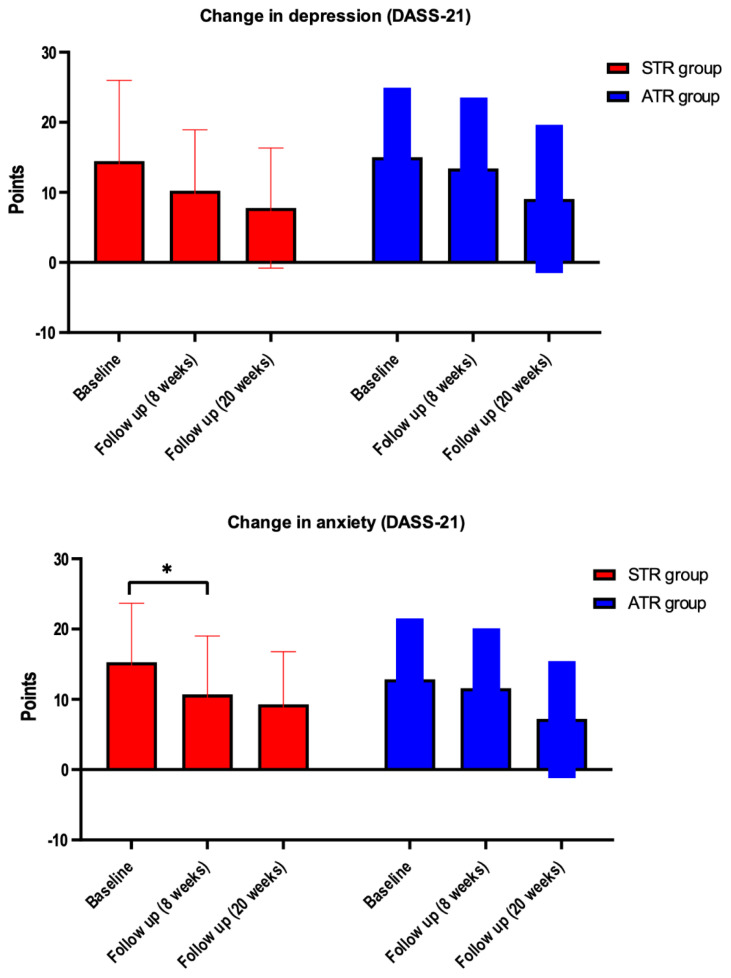
Changes in Depression, Anxiety and Stress Levels Measured by DASS-21 at Baseline, at 8 Weeks of Follow Up and During a Follow Up of 20 Weeks *Note*. Changes within groups in patients allocated to STR group compared to patients allocated to the ATR group; *significant difference (p<0.05) within groups Tukey’s post hoc test

There was no significant difference between groups, but a significant intra-group difference was found in the stress (F = 9.944; *p =* 0.0006), anxiety (F = 9.608; *p =* 0.0015), and depression (F = 7.693; *p =* 0.0028) subscales of the DASS-21 scale. In the post hoc analysis, the difference was found to be between the initial assessment vs. eight weeks of intervention for the STR group (95% CI = 0.03745–12.90; *p =* 0.0483); initial assessment vs. 20 weeks of intervention of the STR group (95% CI = 0.4912–15.74; *p =* 0.0336) in the stress subscale; and between initial assessment vs. 20 weeks of intervention of the ATR group (95% CI = 0.5512–10.70; *p =* 0.0270) in the anxiety subscale. No significant difference was found in the depression subscale (Figure 3). The effect size of the DASS-21 intervention for the stress subscale was of medium magnitude (d = 0.71) within groups; for the anxiety subscale, it was of medium magnitude (d = 0.70) within groups; and for the depression subscale, it was of medium magnitude (d = 0.62) within groups.

### Quality of Life

There was no significant difference between groups. A significant intra-group difference was found (F = 10.49; p = 0.0009) in the WHOQOL-Bref questionnaire. In the post hoc analysis, the difference was located between the initial assessment vs. eight weeks of intervention for the STR group (95% CI = −18.96–−2.664; p = 0.0063) and initial assessment vs. 20 weeks of intervention for the STR group (95% CI = −15.29–−2.792; p = 0.0034). The effect size of the intervention was of medium magnitude (d = 0.73) within groups.

### Adverse Effects

At the study’s outset, the mMRC scale was used to ensure exercise safety for PCS-affected individuals. Predetermined adverse events included hospitalization; participants reported events by text. No exercise-related issues occurred.

## Discussion

This study provides evidence that synchronous, physiotherapist-supervised telerehabilitation—comprising aerobic training, muscle strengthening, stretching, joint mobility, and respiratory training—offers more positive clinical outcomes than asynchronous telerehabilitation for individuals with post-COVID-19 syndrome in low- and middle-income countries. Notably, participants in the asynchronous group faced challenges in actively engaging in their own care. The synchronous approach significantly improved lower limb functionality, perceived dyspnea and fatigue, stress levels, and overall quality of life. In contrast, the asynchronous approach only yielded improvements in perceived fatigue and anxiety. Consequently, the effect size for the synchronous model ranged from medium to large for most outcomes within groups, with the only significant difference between the groups being lower limb functionality.

The field of TR for PCS is relatively nascent. Most existing studies have compared TR with traditional face-to-face or no therapy (Martínez-Pozas et al., 2024). Our findings align with a randomized clinical trial by Tahan et al. (2023) which showed that while both modes are effective, synchronous TR has greater positive impacts on physical functionality. This suggests that the TR mode should align with the target population ’s capability to actively participate in their own care. Clinically, our results underscore the importance of physiotherapeutic oversight in treating PCS via TR.

Both groups in this study experienced reduced fatigue, as measured by the FAS—a symptom reported by 90% of the participants (Moore et al., 2013). Few studies have quantitatively assessed this pervasive symptom in PCS. For instance, a trial by del Corral et al. (2023) did not measure the intervention’s impact on fatigue, and the meta-analysis by Ceban et al. (2022) indicated that unresolved fatigue is linked to functional impairment and can persist for an extended period. A study by Nambi et al. (2022) demonstrated that a moderate aerobic and resistance training protocol improved muscular capacity in elderly males with post-COVID-19 sarcopenia compared to a high-intensity approach. Therefore, telerehabilitation focusing on functional exercises and moderate aerobic activity can effectively mitigate fatigue and should be promoted, as the population more readily accepts it than strenuous exercise regimes.

The time needed to complete the FTSTS decreased solely within the synchronous group, which contrasts with a recent Brazilian clinical trial employing an asynchronous telemonitored post-COVID-19 home exercise program (Teixeira et al., 2022). In the latter study, participants received exercise demonstrations and self-guided instructions, sustaining improvements over a 12-week period. This implies that asynchronous formats may be effective in longer-term applications. Nonetheless, for optimizing lower limb functionality, resistance training should be tailored to individual needs, even within TR contexts.

As illustrated in Figure 1, the attrition rate underscores the challenges associated with implementing a TR program in low- and middle-income countries. This challenge was also identified as a limitation in a randomized clinical trial by Teixeira et al (2022) where participants in the asynchronous group exhibited reduced engagement and failed to achieve significant improvements in both primary and most secondary outcomes. Leochico et al. (2020) emphasized the hurdles in introducing TR in the Philippines, a country with a socioeconomic profile similar to that of Brazil, attributing the lack of enthusiasm to the scarcity of relevant data. In contrast, a randomized clinical trial in China demonstrated improvements in functional capacity and lower limb strength using an asynchronous telerehabilitation model delivered via a mobile application, (Li et al., 2022) suggesting that certain countries can effectively harness technology for asynchronous treatment and self-efficacy.

Despite the encouraging results, this study presents limitations that should be considered in the interpretation of the findings. Initially, the absence of personalized physical training prescriptions adapted to the individual capabilities of the participants stands out, as well as the lack of assessment of the participants’ satisfaction with the exercise programs. In addition, the absence of a control group to monitor the natural evolution of post-COVID syndrome limits the conclusiveness of the inferences; however, due to the public health emergency context, ethical considerations justified offering intervention to all groups. Furthermore, remote assessments, given the restrictions imposed by the COVID-19 pandemic, present other limitations, even though they were a viable methodological alternative during that period.

Specifically regarding the ATR group, its methodology has inherent weaknesses, since it was not possible to control variables such as type, intensity, frequency, and adherence to the activities performed. The non-standardized nature of this exposure may have increased, stimulated the variability of stages, and reduced the robustness of comparisons with the STR group, whose intervention was structured and replicable. Therefore, the results of the ATR should be interpreted with caution and considered predominantly exploratory, while the most consistent instructions derive from the STR, which presented greater methodological rigor and, consequently, greater internal validity. Future studies should address these limitations through personalized instructions, in-person assessments whenever possible, and protocols that allow greater control of variables associated with physical activity.

## Conclusions

Synchronous telerehabilitation positively affected lower limb functional capacity, dyspnea, fatigue, self-reported stress, and quality of life. In contrast, asynchronous telerehabilitation only positively affected perceived fatigue and anxiety. By considering the difficulty of telerehabilitation in a low- and middle-income country, synchronous telerehabilitation supervised by a physiotherapist seems more effective than an asynchronous telerehabilitation program in adults with post-COVID-19 syndrome.

In short, a TR offers a safe, accessible, and effective alternative. These results contribute valuable evidence to the growing body of literature and support the integration of TR into routine clinical practice for with post-COVID-19 syndrome management.

## Figures and Tables

**Table 1:** Participant’s Characteristics at Baseline

Variables	Total (n = 31)	Synchronous (STR) group (n = 17)	Asynchronous (ATR) group (n = 14 )	*Difference between groups p*-value
Age in years, mean ± SD	42.48 ± 12.71	45.29 ± 11.60	39.07 ± 13.58	0.050483
BMI, mean ± SD	28.21 ± 6.44	29.49 ± 6.16	26.66 ± 6.66	0.371966
Overweight, n (%)	16 (51.61%)	8 (47.06%)	8 (57.14%)	>0.9999
Obesity, n (%)	8 (25.81%)	6 (35.29%)	2 (14.29%)	0.1573
**Sex, n (%)**				
Female%	25 (80.65%)	15 (88.24%)	10 (71.43%)	0.3173
Male%	6 (19.35%)	2 (11.76%)	4 (28.57%)	0.4142
**Disease severity, n (%)**				
Hospitalization	3 (9.68%)	1 (5.88%)	2 (14.29%)	0.5637
Ward admission	2 (6.45%)	2 (11.76%)	0 (0%)	0.1573
Without hospitalization	26 (83.87%)	14 (82.35%)	12 (85.71%)	0.6949
History of smoking, n (%)	7 (22.58%)	4 (23.5%)	3 (21.43%)	0.7055
**Comorbidities, n (%)**				
Presence	20 (64.52%)	11 (64.71%)	9 (64.29%)	0.6547
Absence	11 (35.48%)	6 (35.29%)	5 (35.71%)	0.7630
**Type of comorbidity, n (%)**				
Neuropsychiatric diseases	11 (35.48%)	4 (23.53%)	7 (50%)	0.3657
Cardiovascular diseases	10 (32.26%)	6 (35.29%)	4 (28.57%)	0.5271
Lung diseases	1 (3.23%)	0 (0%)	1 (7.14%)	0.3173
Diabetes	3 (9.68%)	2 (11.76%)	1 (7.14%)	0.5637
Other	1 (3.23%)	1 (5.88%)	0 (0%)	0.3173
**Length of illness at the start of study, n (%)**				
Less than a month	5 (16.13%)	3 (17.65%)	2 (14.29%)	0.6547
Up to three months	6 (19.35%)	5 (29.41%)	1 (7.14%)	0.1025
Up to six months	3 (9.68%)	3 (17.65%)	0 (0%)	0.0833
More than six months	17 (54.84%)	6 (35.29%)	11 (78.57%)	0.2253
**Persistent symptoms, n (%)**				
Fatigue	28 (90.32%)	16 (94.12%)	12 (85.71%)	0.4497
Myalgia	13 (41.93%)	7 (41.18%)	6 (42.86%)	0.7815
Arthralgia	16 (51.61%)	7 (41.18%)	9 (64.29%)	0.6171
Other (dyspnea, chronic cough, anosmia, cognitive disorders, hyperacusis, amaurosis)	17 (54.84%)	10 (58.82%)	7 (50%)	0.4669
**Outcomes, mean ± SD**				
FTSTS	11.79 ± 2.89	11.02 ± 2.48	12.62 ± 3.15	0.613519
FAS	33.65 ± 5.49	33.29 ± 4.48	34.07 ± 6.67	0.805476
**DASS-21**				
Stress	19.10 ± 9.63	19.76 ± 9.11	18.29 ± 10.52	0.642618
Anxiety	14.19 ± 8.36	15.29 ± 8.36	12.86 ± 8.47	0.443213
Depression	14.71 ± 10.60	14.47 ± 11.52	15 ± 9.79	0.867102
WHOQOL-Bref	63.8 ± 9.7	62.05 ± 9.4	64.75 ± 10.25	0.394297

*Note*. FTSTS, Five Times Sit-To-Stand; mMRC, Modified Medical Research Council Scale; FAS, Fatigue Assessment Scale; DASS-21, Depression, Anxiety, and Stress Scale-21; WHOQOL-Bref, World Health Organization Quality of Life-BREF

**Table 2:** Change in Lower Limb Functionality, Dyspnea, Fatigue, Stress, Anxiety and Depression Levels, and Overall Quality of Life Variables at Baseline, 8 weeks and During Follow-up

	Baseline	Follow-up weeks)	(8 Follow-up (20 weeks)	Within groups p-value and Cohen’s d (90% CI)	Between groups p-value and Cohen’s d (90% CI)
*Primary outcome*	Mean±SD	Mean±SD	Mean±SD		
**FTSTS**					
Control	12.62±3.15	12.67±4.17	12.01±3.16	*p=*0.0115* d=0.51	*p=*0.0269* d=0.52
Intervention	11.02±2.48	9.63±1.93	9.40±1.83		
*Secondary outcomes*	Mean±SD	Mean±SD	Mean±SD		
**mMRC**					
Control	1.79±0.80	1.43±1.09	1.75±0.89	*p=*0.0123** d=0.54	*p=*0.6672 d=0
Intervention	1.94±0.66	1.06±0.97	1.53±0.92		
**FAS**					
Control	34.07±6.67	29.79±7.86	18.08±16.24	p<0.0001*** d=1.06	*p=*0.8244 d=0
Intervention	33.29±4.48	24.06±6.81	22.94±10.97		
**DASS-21**					
**Stress**					
Control	18.29±10.52	17.71±10.04	10.46±9.80	*P=*0.0006*** d=0.71	*P=*0.7398 d=0
Intervention	19.76±9.11	13.29±9.03	11.65±9.68		
**Anxiety**					
Control	12.86± 8.47	11.57±8.31	7.23±8.10	*p=*0.0015** d=0.70	*p=*0.6451 d=0
Intervention	15.29± 8.36	10.71±8.30	9.29±7.48		
**Depression**					
Control	15±9.79	13.43±9.94	9.08±9.08	*p=*0.0028** d=0.62	*p=*0.5821 d=0
Intervention	14.47±11.52	10.24±8.69	7.76±8.57		
**WHOQOL-Bref**					
Control	64.75±10.25	67.64±8.51	61.73±9.59	*p=*0.0009*** d=0.73	*p=*0.2997 d=0.09

Intervention	62.05±9.4	72.85±13.78	71.08±11.11		

*Note*.

* Statistically significant differences p<0.05;

** Statistically significant differences p<0.01;

*** Statistically significant differences p<0.00;

FTSTS, Five Times Sit-To-Stand; mMRC, Modified Medical Research Council Scale; FAS, Fatigue Assessment Scale; DASS-21, Depression, Anxiety, and Stress Scale-21; WHOQOL-Bref, World Health Organization Quality of Life-BREF

**Table 3 t3-ijt-17-2-6716:** Telerehabilitation Exercise Protocol in Both Groups

	Duration (seconds)	Final intensity
**Heating**		
Stationary march	60	BORG 6
Jumping jacks	60	BORG 6

**Global stretches**		
Pectoralis major	60	Maximum stretch
Posterior chain of lower limbs	30	Maximum stretch
Quadriceps	60	Maximum stretch
Pectoralis minor	60	Maximum stretch
Gastrocnemius and soleus	60	Maximum stretch
Chest and upper limbs with maximum sustained inspiration	30	Maximum stretch

	**Series x repetitions**	**Rest**

**Joint mobility**		
Spine in flexion and extension	1x10	10 seconds
Thoracic rotation and extension	1x10	10 seconds

**Breathing exercises**		
*Labial frenum	3x6	30 seconds

	**Series x repetitions**	**Rest**

**Lower limb strengthening**		
Sitting and standing	3 x 8**	60 seconds
Squats	3 x 8**	60 seconds
Hip flexion and extension	3 x 8**	60 seconds
Bridge	3 x 8**	60 seconds
Plantiflexion	3 x 8**	60 seconds

**Upper limbs strengthening**		
Biceps	3 x 8**	60 seconds
Triceps	3 x 8**	60 seconds
Support	3 x 8**	60 seconds

**Abdominal strengthening**	3 x 8**	60 seconds

* Recommended every day at home and unsupervised in both groups.

** Increase by two repetitions every two weeks.
